# Automated Exploration of Prebiotic Chemical Reaction Space: Progress and Perspectives

**DOI:** 10.3390/life11111140

**Published:** 2021-10-26

**Authors:** Siddhant Sharma, Aayush Arya, Romulo Cruz, Henderson James Cleaves II

**Affiliations:** 1 Blue Marble Space Institute of Science, Seattle, WA 98154, USA; sharmasi@chalmers.se (S.S.); aayush.11912610@lpu.in (A.A.); romulo.cruz.s@uni.pe (R.C.); 2 Department of Biochemistry, Deshbandhu College, University of Delhi, New Delhi 110019, India; 3 Department of Chemistry and Chemical Engineering, Chalmers University of Technology, SE-412 96 Gothenburg, Sweden; 4 Department of Physics, Lovely Professional University, Jalandhar-Delhi GT Road, Phagwara 144001, India; 5 Big Data Laboratory, Information and Communications Technology Center (CTIC), National University of Engineering, Amaru 210, Lima 15333, Peru; 6 Earth-Life Science Institute, Tokyo Institute of Technology, Tokyo 152-8550, Japan

**Keywords:** prebiotic chemistry, automated chemical space searches, chemical reaction networks, computational modelling, network autocatalysis, self-replicating structures

## Abstract

Prebiotic chemistry often involves the study of complex systems of chemical reactions that form large networks with a large number of diverse species. Such complex systems may have given rise to emergent phenomena that ultimately led to the origin of life on Earth. The environmental conditions and processes involved in this emergence may not be fully recapitulable, making it difficult for experimentalists to study prebiotic systems in laboratory simulations. Computational chemistry offers efficient ways to study such chemical systems and identify the ones most likely to display complex properties associated with life. Here, we review tools and techniques for modelling prebiotic chemical reaction networks and outline possible ways to identify self-replicating features that are central to many origin-of-life models.

## 1. Introduction

The study of prebiotic chemistry requires understanding complex phenomena involving the interplay of highly variable and as-yet uncertain primitive environmental conditions, often in the context of diversity-generating chemical reactions [1]. These reactions may have together produced large and diverse sets of products that can differ subtly or dramatically under variable conditions, e.g., [2,3,4,5]. This interplay has been speculated to have produced the emergent chemical systems which gave rise to life. However, the specific environmental conditions and chemical processes which gave rise to life have now been lost to Earth’s dynamic geological history, and it is difficult for experimentalists to recreate all possible combinations of conditions that may have been present on primitive Earth in the laboratory, or analyze the complex products which often result from such lab simulations [6].

Computational approaches (see for example [7]) offer efficient ways for chemists to study chemical systems which may display complex properties conducive to the emergence of chemical systems with life-like properties. Here we name the putative collection of chemical processes, their interplay with environmental parameters, and the resultant chemical diversity that appears in such a computational model as a chemical reaction network representation (CRNR). CRNRs may be thought of as idealized collective material flows through allowed reactions channels, which can vary as a function of reaction conditions, including temperature, pH, concentration, molecularity, etc. (e.g., [8,9,10]).

**Figure 1:**
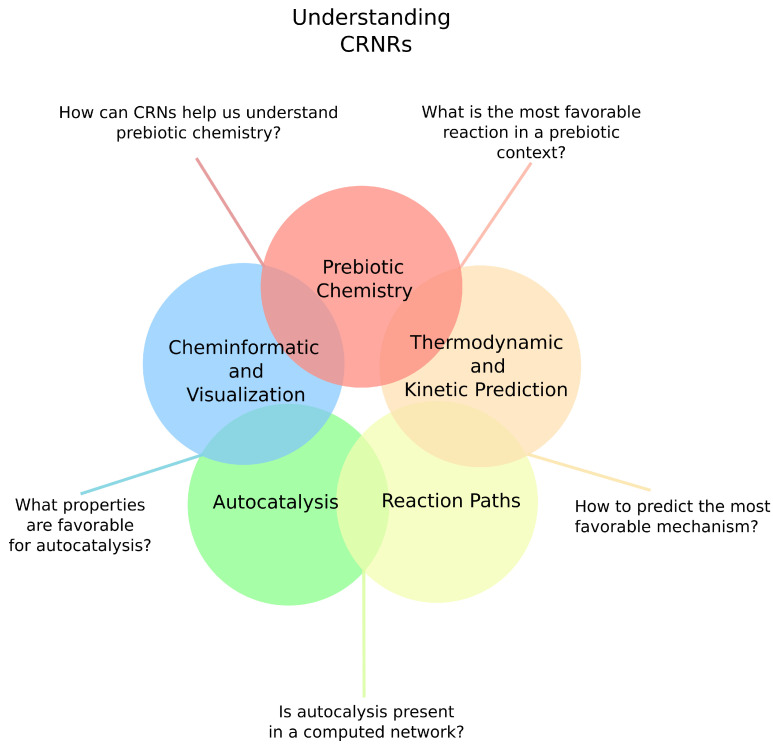
Some of the current questions and methods for the exploration of prebiotic CRNRs.

These representations may not necessarily be complete descriptions of complex reaction systems, but may nonetheless offer roadmaps for understanding complicated fluxes through chemical systems. Many aspects of real chemical reaction network (CRN) chemistry may not be realized in CRNRs. Understanding why CRNRs fail to accurately mimic real CRN outcomes is a central challenge for computational chemistry to help understand prebiotic chemistry and the origins of life, and offers a route to improve the use of CRNs as guides for such purposes. Many important questions remain as to how real CRNs could have become capable of Darwinian selection [11,12]. Some authors have suggested the emergence of complexity due to network properties may be as important as the nature of the chemical reactions involved in CRNs [13,14]. An overview of current questions and methods aiding in the exploration of prebiotic chemical reaction space is depicted in Figure 1.

**Figure 2:**
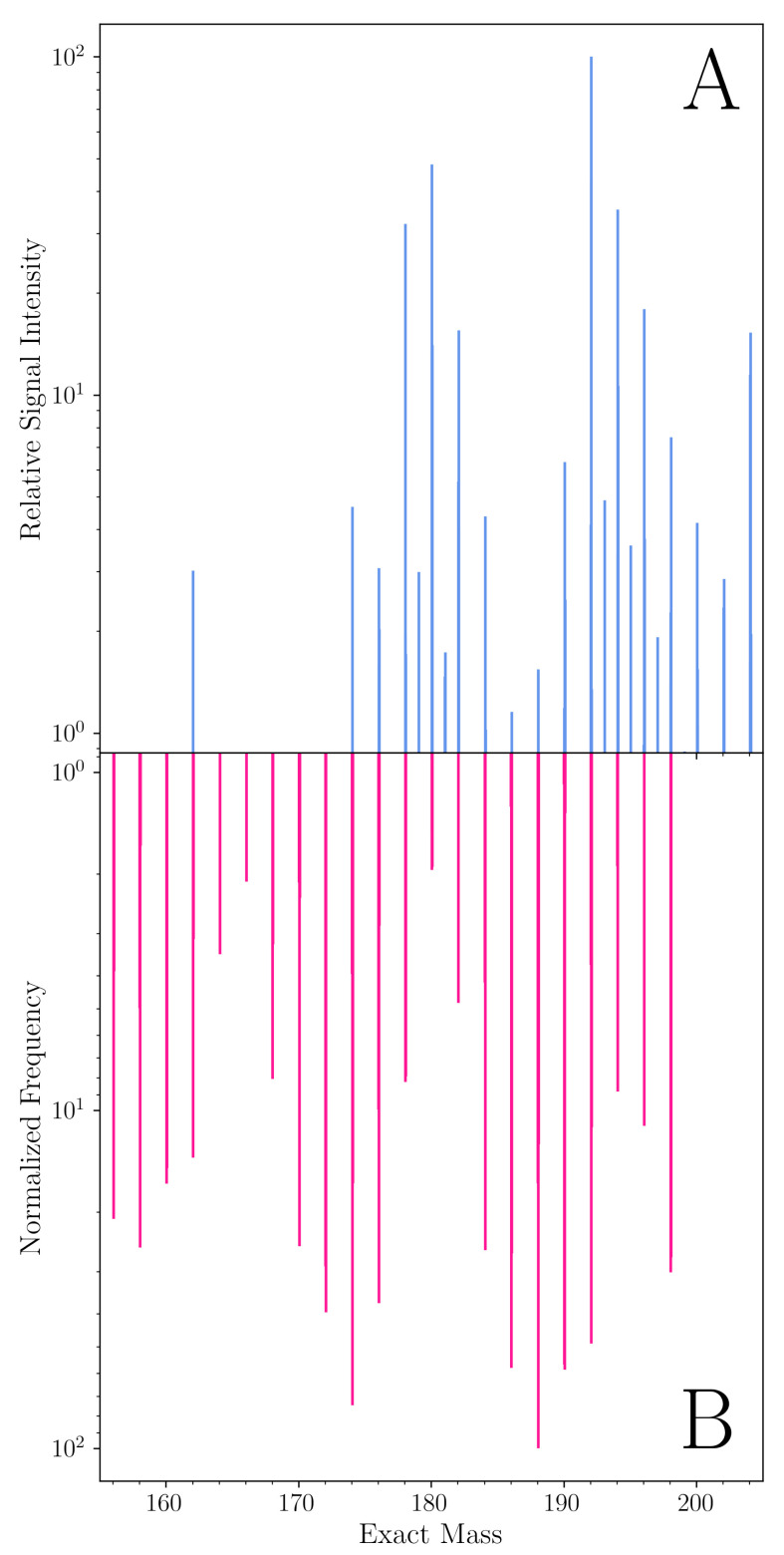
CRNRs may accurately predict the many features of CRNs. (**A**) The 150–210 amu region of the mass spectrum of the products of a laboratory formose reaction measured using high-resolution Fourier transform ion cyclotron resonance mass spectrometry (FT-ICR-MS) in negative ESI mode. (**B**) The predicted mass distribution after six generations of the products of the same reaction was generated using CRNR methods. In this formose reaction, 2 M paraformaldehyde, 1 M glycolaldehyde, and 0.05 M Ca(OH)${}_{2}$ were heated in aqueous solution in sealed glass ampoules under nitrogen at 85° for eight days. The code for generating this figure is described in Supplementary Materials.

Comparing computational and experimental investigations of CRN reactions, real-world reactions may produce hundreds to millions of products, and their identification is limited by analytical method detection limits. The size of the product space that can be practically computed, if not carefully informed by undetermined variables associated with kinetic parameters, may also grow exponentially (e.g., [15]). Locally variable environmental factors, such as the presence of certain minerals (e.g., [2,16]), may also alter the course of reactions and steer product distributions (e.g., [17]). Figure 2 illustrates one example of how CRNRs attempt to predict the outcomes of real-world CRNs.

Because the salient features of prebiotic chemical systems may be difficult to measure directly, CRNR methods [18,19], in silico exploration of high-dimensional chemical spaces (e.g., [15,20,21]), and network theory offer promising tools to explore origins questions (e.g., [12,22]). As Smith et al. [23] have pointed out, network theory may be useful in the study of collective chemical behavior. It is crucial that collective systemic behavior be understood to fully characterize a chemical or biological system, as the study of only one or a few components of a complex product suite is likely inadequate to infer higher-order properties of chemical systems.

A large variety of computational tools can be pipelined in CRNRs to explore prebiotic chemical reaction space, ranging, for example, from ab initio molecular dynamics (MD) simulations (e.g., [24,25]) to chemical assembly theory (e.g., [26]), and molecular assembly trees (e.g., [27]). Informatics has also opened many new avenues of study in biology and chemistry [28]. Chemoinformatics has rapidly become a routine discovery tool [29], with ever more powerful open-source resources becoming available [30,31]. Likewise, the types of chemical systems of prebiotic relevance that CRNRs can be applied to are diverse, ranging from experimental methods for life detection (e.g., [32]) to the simulation of primitive planetary atmospheric chemistry (e.g., [33]).

In the present article, we briefly review the developments in computational chemistry that can assist in the application of CRNR computation and analysis to understanding problems of astrobiological relevance, especially prebiotic chemistry.

## 2. Modelling Prebiotic Chemistry: From Individual Reactions to a Network

While performing a bottom-up synthesis of a computational reaction network representation, going from a small set of reaction species to a network often requires making approximations due to time and computing resource limits. One must be wary of the sacrifice in the accuracy of model predictions when such approximations are made. The advantages of the different approaches to modelling, from quantum chemistry to graph theory, must be weighted based on the scale of the network and the desired accuracy. For example, rigorous quantum chemistry can provide a more precise estimation of the mechanism, kinetics or outcome of a single reaction, while graph theory-based modelling, with its less costly computations, can provide a convenient framework for the synthesis, visualization and analysis of large scale CRNRs from a network theory perspective. Here, we contrast some of the approaches that have gained traction in prebiotic chemical modelling.

The first of these sets of approaches use quantum chemistry-based computations, which can provide accurate predictions of reaction outcomes (e.g., [34]). Computational quantum chemical approaches have been used to understand prebiotic reaction pathways (e.g., [35,36,37]); however, these approaches often scale poorly due to the cost of computation involved, which currently limits their use in simulating complex prebiotic networks [38]. Besides computational resource issues, such approaches may rely on prior knowledge of intermediate transition states or efficient searches for these intermediates on the reaction’s potential energy surface (PES) (e.g., [39]). More recently, computational efficiency of the global optimization problem algorithm has been explored by combining quantum chemistry techniques with network analysis methods such as exploration of optimal thermodynamic and stoichiometric pathways [40] or random sampling processes [34].

**Figure 3:**
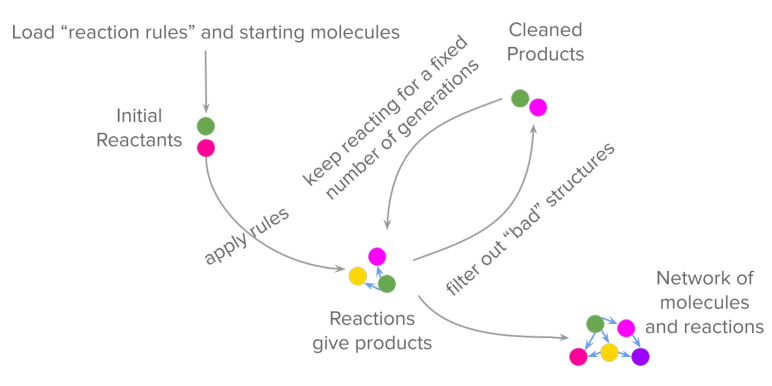
Schematic for the forward synthesis of a network using graph theory-based tools. Typically, a set of “reaction rules” are loaded, which specify how certain substructures are to be altered during a reaction. All the rules are then applied combinatorially to a set of initial reactants, which gives a set of products as the graph transformations happen that give product molecules, connected by “edges” representing reactions. Constraints that certain molecules (or “graphs”) with certain substructures should not form can be used to filter out unstable species. Iterative application of these rules to the product suite at each step gives a complete CRNR.

Chemical graph theory approaches, on the other hand, offer a way to streamline handling of large CRNRs [41,42]. Graph theory is commonly used in computational chemistry since molecules can be precisely represented as graphs [43], with nodes representing atoms and edges representing chemical bonds between them. These molecular “graphs” are then transformed by applying user-defined reaction templates that guide the synthesis of products using “seed” molecules. The reaction templates are rules that search for a particular pattern in a molecule, then apply transformations by modifying the edges of the graphs. The accuracy of reactions performed this way thus has a clear dependence on the selected reaction mechanism, and can be tuned to be appropriately restrictive or permissive. For more reliable reaction predictions using graph theory, one can use estimates of thermodynamic parameters to determine the feasibility of a reaction. A schematic describing how multiple rounds of reaction generation leads to the synthesis of a full network is shown in Figure 3.

**Figure 4:**
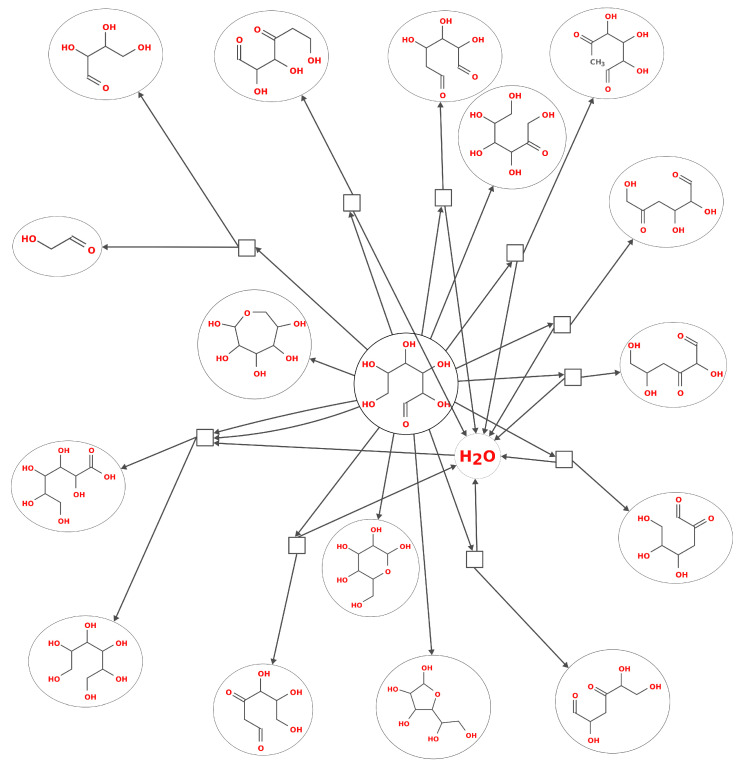
A primitive CRNR for glucose degradation reaction, generated using the graph grammar-based program MØD. The in silico synthesis of this CRNR was done as per the general methods outlined in Figure 3. The reaction rules used were selected based on prior knowledge of mechanisms known to dominate in this chemical system under pH and temperature conditions of interest. A single cycle of reaction rule application (i.e., one “generation”) is shown here. In the visual representation used here, molecules are shown in ovals, while reaction nodes are shown as squares.

Graph theory-based tools have been developed to use intuitive ways of representing molecules (e.g., simplified molecular input line entry system, SMILES) and codifying reactions, for instance, the human-readable Graph Modeling Language (GML) format used in tools such as MØD, a software package developed for graph-based cheminformatics [41]. Graph theory also allows for various generalizations of sophisticated chemical phenomena, for example, allowing concepts such as autocatalysis and tautomerism, among others, to be formalized. Graph grammars can encode generalizable reaction mechanisms (referred to in Figure 3 as “reaction rules”). An early stage CRNR generated for the glucose degradation reaction using the graph-grammar approaches is shown in Figure 4.

Various tools for constraining the feasibility of reactions using thermodynamic calculations based on quantum chemistry methods exist. Quantum chemical calculations have been employed to provide more accurate determinations of the course of reaction pathways in prebiotic reaction networks [44]. Density functional theory (DFT) methods have been used to characterize thousands of molecules and chemical reactions [45] but are expensive in terms of required computing resources. However, there are classical group contribution methods (GCMs) that are less computationally demanding [46]. Joback and Reid’s classic GCMs can be used on the chemical reaction spaces with the help of tools such as JRgui [47], eQuilibrator [48] and the Benson group’s additivity methods [49]. The eQuilibrator program makes use of component contribution methods, which are a modification of GCM methods [50]. Semi-empirical approaches to studying prebiotic chemical thermodynamics have been made using software packages such as MOPAC [51]. Automated approaches like AutoMeKin combine molecular dynamics (MD), graph theory algorithms and Monte Carlo simulations to discover likely relevant reaction mechanisms [52]. Quantum mechanics/molecular mechanics (QM/MM) and MD simulations have been combined to study prebiotic nucleic acid analogues [53] and lipids [54]. Kua et al. [55], benchmarked thermochemical estimations for compounds thought to be important in protometabolism derived from eQuilibrator to those derived from more accurate yet computationally demanding DFT quantum chemical methods, and found them to be remarkably similar.

Interactive frameworks for exploring chemical reaction space have been developed, for example Molpher software [56]. Bespoke automated computational approaches for generating CRNRs can be constructed modularly, for example by integrating graph grammar operations such as those used in MØD [41], reactive molecular dynamics tools such as ReacNetGenerator [57] and Python (programming language frameworks) such as Reaction Mechanism Generator (RMG) [58,59], CGRtools (Condensed Graph of Reaction) [60], and Rule Input Network Generator (RING) for generating CRNRs from complex reactive systems [61], among others. Other tools potentially useful for constructing such pipelines include pReSt [62] to discover novel chemistries in automated CRNRs, and CERENA (ChEmical REaction Network Analyzer) [63] to model stochastic chemical kinetics in chemical reaction networks.

For the sake of simplicity, many computational studies relying on graph theory use flattened molecular representations that lack stereochemical information, but stereochemical information can be encoded in such frameworks [64]. Such information can be used to gain insight into the kinetic mechanisms of stereochemical symmetry breaking which evidently occurred during the emergence of homochiral biological systems [65]. Efficient stereochemical handling in chemical reaction networks is so far limited to relatively small systems [66], partially due to complexities in representing Cahn–-Ingold–-Prelog rules to unequivocally label stereoisomers. The ability to more agiley model stereochemical transformations would be a major advance for this field. Open source stereoisomer generation methods include RDKit’s EnumerateStereoisomers module [67] and MAYGEN [68], among many others.

## 3. Detection of Autocatalytic Motifs in Computed Chemical Networks

A common feature of origins of life models is the involvement of self-replicating molecules or systems [69,70,71,72,73,74], which can be quantified as autocatalytic sets within CRNs [75]. Autocatalysis represents a range of phenomena of variable complexity potentially responsible for many processes of prebiotic interest [76], for example the formose reaction e.g., [77] and formaldehyde-catalyzed HCN oligomerization [78]. The detection of the emergence of autocatalysis is a major goal of prebiotic chemistry [79], and autocatalytic reactions may represent an important link between prebiotic chemistry, primitive metabolism and modern biochemistry [80,81,82]. It remains to be determined how common autocatalytic reaction systems are; they may be rather common but simply hard to detect, depending on their kinetics, complexity and context, or truly relatively rare.

There are two fundamentally different types of autocatalytic networks: ones which produce autocatalysis through topological network effects and those which generate feedback catalysis [83], in which some products of the reaction networks serve as true catalysts (as opposed to network catalysts) for existing or novel reactions. Both types can potentially fundamentally change the evolution of CRNs [79,84,85].

**Figure 5:**
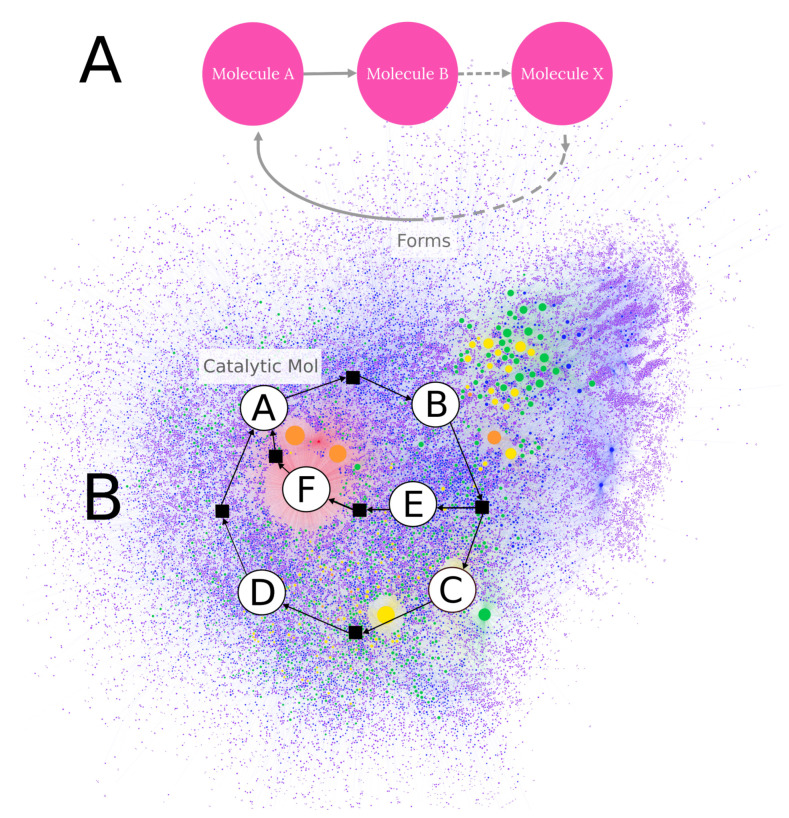
General features of autocatalysis and a specific example of an autocatalytic cycle detectable within CRNRs. (**A**) The basic idea of autocatalysis is that a sequence (or network) of reactions begins with a specific molecule A and produces more than one copy of A, assuring that the cycle produces more A than it consumes. (**B**) A concrete example of such a cycle within a larger CRNR. Here, two paths, A→B→E→F→A and A→B→C→D→A, contribute to produce stoichiometrically larger quantities of A. The CRNR illustrated here was produced using five rounds of reaction generation in the glucose degradation chemistry discussed above. The layout of the graph was executed using Gephi. The size of the nodes corresponds to each node in-degree. Each color represents a new generation.

The former type depends most heavily on thermodynamic considerations: in order for a large number of reactions to proceed favorably and at a comparable pace, they must have low activation energy barriers. Indeed a frequent aspect of many proposed prebiotic chemical reactions is their seeding by high free-energy species generated by the action of environmental energy sources (e.g., HCHO or HCN generated by electric discharges, photochemically, etc.). CRNs can be driven by various energy inputs (e.g., [9]), including radioactive decay [86,87,88,89], though it might be expected that solar radiation may be more important due to its larger flux [90], and the dissipation of potential chemical energy likely plays a role in the development of CRNs. Dissipative chemistry may partially explain the growth of complex CRNs [91]. Semenov et al. [92] studied a nonenzymatic autocatalytic reaction network using continuous st flow reactors and found them to display oscillatory behavior. CRNRs may offer methods for designing such chemical systems rapidly from first principles. An autocatalytic loop motif in a sample CRNR is illustrated in Figure 5.

Andersen and colleagues [93] have proposed the use of integer hyperflows, which explore how varying stoichiometric relationships among CRN pathways may affect the overall flux of material through them as tools for the universal definition of autocatalysis in chemical reaction networks. Chemical cycles such as the reverse tricarboxylic acid cycle have been explored computationally [94,95], and graph grammar methods can also be applied to study alternative prebiotic pathways such as Eschenmoser’s glyoxylate pathway [96].

## 4. Use of Machine Learning (ML) for Understanding CRNs

ML has transformed computational chemistry and also holds immense potential for exploring chemical systems [97,98]. ML and neural networks (NNs) have been used to explore molecular structures, reactions and reaction mechanisms (e.g., [99]), and these methods can be applied for exploration of the generated CRNRs. The number of chemical reactions or transformations a molecule can participate in can be examined using automated reaction template analysis [100]. Much like graph theory-based implementations, deep learning has been applied to generating molecular structures and predicting their properties [101]. The use of ML in cheminformatics to predict plausible reaction mechanisms (e.g., [102,103]) has allowed approaches such as NN-based methods to predict chemical reaction space [104]. Combined graph theory-NN methods have also been used to predict the activation energies of organic reactions [105].

ML methods have been coupled with chemoinformatic molecular descriptors [106] and structure-based reactivity estimation approaches to predict reaction outcomes [107,108]. Deep learning, which is a subset of ML, has been used in chemical reaction prediction [109] and to predict reaction yields [110] using interfaces like IBM RXN for Chemistry [111,112], which can be further modified to predict enzymatic reactions [113], and being open-source, these approaches can be readily modified to meet user requirements. Meuwly [114] reviewed the utility of ML methods for chemical reactions. To date, we are not aware of careful comprehensive comparisons of these methods which would suggest one approach is better than another, merely that applying such approaches culls CRNR outputs.

## 5. Problem-Specific Cheminformatic Tools and Approaches

### 5.1. Computing Molecular Descriptors

It is often useful to characterize chemical species quantitatively using molecular structure based metrics. “Molecular descriptors” estimate properties such as octanol/water partition coefficients (LogP), aqueous solubility [115], and drug-likeness [116], among various other properties including topological and geometric ones. Computed descriptors provide a way to compare a wide range of species with varied structural and chemical properties, and to identify particular molecules with certain desirable properties or collections of properties among very large datasets. Descriptor-based analyses are often used for chemical space exploration, e.g., [117]. Tools for descriptor computation include PaDEL [118], RDKit [119], ChemDes [120,121], Mordred [122], CDK-GUI [123] and PyBioMed [124]. There is some overlap with respect to the descriptors each of these packages computes, in addition to unique functionalities of each package.

### 5.2. Broad Functionality Chemoinformatics Tools

RDKit [119] is a widely used general purpose cheminformatics package with functionalities for molecular manipulation, curation, library building, and molecular analysis that also computes numerous molecular descriptors supporting various cheminformatics input formats such as SDF, SMILES, MOL and reaction templates such as SMARTS and SMIRKS [125]. Similar toolkits exist which have at least some degree of overlap in functionality, such as CDK [123], Indigo Toolkit [126], and OpenBabel [127], and are available as a bundle within a single package called Cinfony [128], which provides a simplified programmable application programming interface (API) for cheminformatic operations, and Chembench [129], which is a publicly accessible cheminformatics web portal to mine and model chemical data.

### 5.3. Handling Isomerism

Representations of molecular structures are approximations of real chemical bonding, and chemists have developed several concepts, such as resonance and tautomerism, to deal with nuances glossed over by these shorthand representations. These phenomena have collectively been termed delocalization-induced molecular equality [130], and the multiplicity of equivalent representations of the same compound can create complications in the computational generation of reaction networks, as they may introduce meaningless redundancies. Tautomerism is a challenging problem in computational chemistry [131] and tautomers may represent unique formalisms for understanding how chemicals engage in reactions, thus making their representation meaningful. There are many open-source software packages and libraries available for treatment of computationally generated tautomers and isomers. Tautomer generation can be accomplished using open-source tools such as AMBIT [132], TautGen [133], and RDKit’s MolVS Wrapper [134], which has been used to enumerate all possible tautomers in small molecule libraries [135]. Each of these has its own unique approaches to enumerate and prioritize tautomeric structures. Commercial software packages, including OpenEye [136], Chemaxon [137] and CACTVS [138], are also able to enumerate and rank tautomeric structures in terms of their relative importance. Databases like TautoBase [139] may also help in the comparison and evaluation of tautomers.

It is often important to find the lowest energy conformers of chemical species to predict energetically plausible reaction mechanisms. Several methods are available to explore PES for this purpose, each with its own nuances and utility. Applications like Molassembler [140] combine molecule generation and conformer exploration methods in a single package. Conformer generation tools using semi-empirical approaches include DataWarrior [141], OMEGA [142], Balloon [143], Confab [144], ConfGen [145], Frog2 [146] and RDKit, which use various force-field estimations and algorithms [147,148]. Packages employing molecular mechanics such as Tinker 8 [149] have also been used for conformer generation, which allows thorough searches for low-energy conformers [150].

Graph-based conformer clustering methods, such as AutoGraph, are helpful for generating ensembles of lowest-energies conformers after conformers have been processed with semi-empirical methods [151].

### 5.4. Miscellaneous Tools

Libraries such as ChemPy [152] and Catalyst.jl [153] were built to explore the dynamics of chemical reaction networks by solving systems of coupled continuous or stochastic differential equations and handling of chemical kinetics processes. Apart from Python, several cheminformatics tools and libraries have been developed in other languages as well, including, in the R programming language, such resources as ChemMine tools [154], ChemmineR [155] and rcdk [156]. The MolecularGraph.jl package provides cheminformatic capabilities using the Julia language [157].

Platforms like Dask [158] help bring the power of parallel computing to cheminformatics for large-scale, rapid analysis and manipulation of cheminformatic data. Calculated molecular fingerprints are used for rapid substructure matching and similarity searching [159] and can be calculated with tools like chemfp [160]. Molecular Set Comparator [161] uses cheminformatic approaches to compare two sets of datasets and properties like Tanimoto distances [162] are used to compare similarities in molecular representations as reduced to a two-dimensional mapping using t-distributed stochastic neighbor embedding (t-SNE, [163]). Statistical tools like principal component analysis (PCA) can also help in similarity clustering, which can be useful for identifying where similar types of species are generated among CRNs. Various visualization tools, including chemical scaffold networks and trees, can be generated and analyzed using automated scaffold graphs [164] or deep learning methods [165]. These may assist in the interpretation of the organization of these features as they are generated in CRNs.

Generating pKa data for organic molecules can be done by using proprietary software such as ChemAxon, and there has been considerable interest in computationally predicting pKa values, either by using ML methods coupled to QSAR (Quantitative Structure-Activity Relationship) [166,167] or by using Graph NN methods [168], developing interactive applications such as MolGpka [169].

Stoichiometric network analysis and flux balance analysis for such networks [170,171] can help get kinetic information off the reactions participating in the network.

## 6. Experimental Vetting of the Computational Methods

Experimental validation of computational models can be accomplished through comparison with products detected using chemical analysis [172,173], for example by using integrated NMR and mass spectral approaches [174,175,176], which provide complementary information regarding chemical diversity and bonding. Van Krevelen diagrams are used by geochemists to characterize large sets of chemical species by plotting the atomic ratios of certain elements. Automated R and Python libraries exist that can generate Van Krevelen diagrams from MS data [177,178]. Kendrick mass defect (KMD) analysis has been used to study large chemical networks using FT-ICR-MS data [32,179], allowing easy identification of homologous series of molecules. R programming language libraries to rapidly analyze such data to generate Kendrick mass defect (KMD) diagrams are commonly used [180]. To see if a CRNR is able to synthesize known metabolites, it may be useful to refer to databases like REAXYS [181], the Human Metabolites Database (HMDB, [182]), and the Kyoto Encyclopedia of Genes and Genomes (KEGG, [183]). The R package biodb [184] provides an interface for querying such chemical databases, while open source packages like Webchem enable scraping of several databases of interest [185]. Similar software packages are available to query these databases, such as PubChemPy for PubChem [186]. Many of the compounds and reactions submitted to the databases discussed in this section are biologically, medically, or industrially relevant. There is a need unified for libraries and databases of prebiotic relevance that accurately predict compounds that should be expected to occur in abiotic systems. Creating databases specific to prebiotic considerations would help train the data used in Machine Learning (ML) models.

## 7. Visualization of Chemically Relevant Datasets

**Figure 6:**
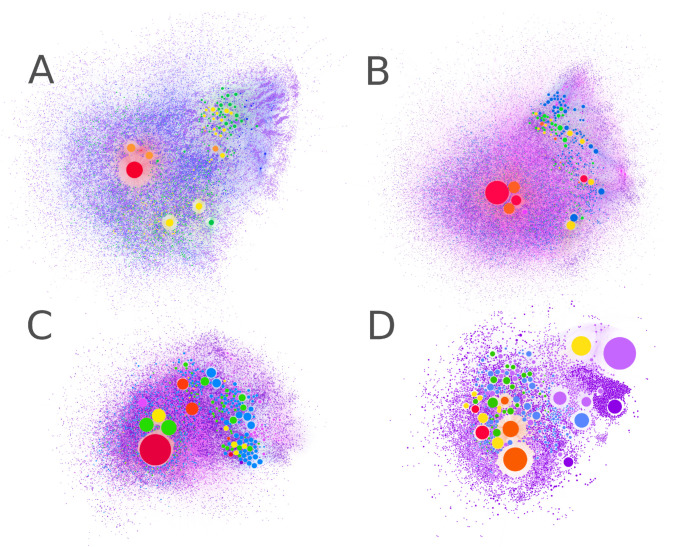
Gephi representations of CRNRs for (**A**) glucose degradation, (**B**) formose reaction, (**C**) pyruvic acid degradation, and (**D**) the reaction of HCN + NH_3_. All CRNRs were generated using the graph-grammar techniques previously discussed, and the figures shown here were produced using the Gephi software. Such visualization tools are helpful for broad visual classification of CRNRs. The size of the nodes in these graphs is proportional to the node’s in-degree as a function of how many edges reach it. Node color indicates the generation in which each compound was first generated. The code for generating this figure is described in Supplementary Materials.

Visualizing dense datasets is a challenge in computational chemistry [187] and there are various interactive approaches for displaying “big data”, such as the use of minimum spanning trees in TMAP [188] and WebMolCS [189] for interactive visualization of chemical space. CRNRs can also be visualized using Gephi [190] (see Figure 6). To visualize networks using Gephi, firstly, a “source-target” table is required that lists how the nodes are connected, indicating which reactive node or product of a certain reaction is relevant. Subsequently, a layout based on the Gephi Force-Atlas 2 algorithm is made to show how to group the clusters according to its connectivity. Some additional tools that are used to visualize CRNRs are CytoScape [191], NetworkX [192], Graph Tool [193] and ReNView [194].

Graph databases such as Neo4J [195] can aid in developing pattern searching algorithms, along with enhanced visualizations of the reaction sequences generated. The associated SMILES format for cheminformatics can be visualized and embedded using tools such as SmilesDrawer [196] and Leruli [197], which help in the development of web-based representations of datasets for easier community access.

## 8. Conclusions

There has been considerable progress in computational methods to predict the outcomes of organic reactions. Detailed and accurate modelling procedures exist for the prediction of most single-step reactions and for reactions with ambiguous mechanisms. However, applying such techniques to concatenated reactions is often resource-expensive and difficult to scale to the degree that it would be of benefit to prebiotic chemists. Less sophisticated techniques involving lesser computational costs, such as those based on graph theory, exist, but they come at the expense of reliability of the predictions, as they are not based on estimates of some physical or chemical parameters that drive reactions.

To accommodate the needs of prebiotic chemists, it is likely preferable to employ a blend of chemical graph theory that is guided by thermodynamic energy estimates to determine reaction feasibility using semi-empirical methods (e.g., the eQuilibriator API). Such semi-empirical methods provide a good approximation consistent with low-level quantum mechanical treatments. Having generated a network, one can study the essential features of the chemistry that is involved. In this context, being able to identify self-replicating features such as autocatalytic cycles could be useful for prebiotic chemists. Although an agreed-upon definition of autocatalysis remains to be defined, it is possible to detect topological features in networks that one considers to resemble autocatalysis.

Studying large networks and analyzing their product suites manually can be difficult. One can make use of statistical metrics to quantify broad, important features of the network as a whole, or find clusters within the network. Additionally, one can make use of molecular descriptors to quantitatively assess structural properties of the products of the chemistry and see how they vary within the network. As for any scientific model, the ultimate test for these computer models is an agreement with the experiment. One ought to test if the CRNR predictions can match with observations, which could be gathered using mass spectrometry or analytical detections of experimental syntheses.

There are many potential applications of these computational tools to astrobiology, including understanding prebiotic chemistry and the origins of life. In addition to concerted studies to increase the accuracy of prediction methods, the development of user-friendly open-source pipelines will allow much greater community development of methods for rapidly exploring prebiotic chemistry in silico to understand real-world phenomena.

## Data Availability

Not applicable.

## Supplementary Materials

The code for generating Figure 2 and Figure 6 is available online on GitHub: https://github.com/ssiddhantsharma/sharmaaryacruzcleaves2021.
